# Comparison of Tick Control and Antibiotic Use Practices at Farm Level in Regions of High and Low Acaricide Resistance in Uganda

**DOI:** 10.1155/2020/4606059

**Published:** 2020-08-27

**Authors:** Joseph Byaruhanga, Fred Odua, Yvette Ssebunya, Olivia Aketch, Dickson Stuart Tayebwa, Innocent B. Rwego, Patrick Vudriko

**Affiliations:** ^1^Research Center for Tropical Diseases and Vector Control, Department of Veterinary Pharmacy, Clinics and Comparative Medicine, School of Veterinary Medicine and Animal Resources, College of Veterinary Medicine, Animal Resources and Biosecurity, Makerere University, Kampala, Uganda; ^2^Division of Veterinary Regulation and Inspection, Department of Animal Health, Ministry of Agriculture, Animal Industry and Fisheries, Entebbe, Uganda; ^3^One Health Division, Department of Veterinary Population Medicine, College of Veterinary Medicine, St. Paul, Falcon Heights, MN 55108, USA; ^4^Department of Biosecurity, Ecosystems and Veterinary Public Health, College of Veterinary Medicine, Animal Resources and Biosecurity (COVAB), Makerere University, Kampala, Uganda

## Abstract

Uganda has experienced tick acaricide resistance in the livestock sector. With increase in incidence of tick-borne diseases (TBDs), use of antibiotics for control of TBDs and other opportunistic diseases has raisedserious concerns. The purpose of this study was to compare the farmers' tick control and antibiotic use practices on farms in regions of low (LARA) and high (HARA) tick acaricide resistance in Uganda, determine the prevalence of antibiotic residues in milk from both regions, and identify factors associated with antibiotic residues in milk. One representative district was selected from each region from which 10 farms were randomly selected. Delvotest SP-NT® test kit was used to detect antibiotic residues in milk. Half-body tick counts and acaricide efficacy tests were performed. Majority (70%) of HARA's respondents reported a corresponding increase in a monthly incidence of TBDs with an average of 3.2 cases of TBDs treated per farm compared to 0.2 cases in LARA. East Coast fever (ECF) was identified as the most common TBD in both regions, though cases of coinfection were more common in HARA. Half of HARA's respondents reported a corresponding increase in the use of antibiotics on their farms due to tick resistance compared to LARA. Antibiotics were the most used drugs on farms in both regions with oxytetracycline being the commonly used antibiotic. Ticks from HARA were resistant to deltamethrin, amitraz, and coformulation (chlorpyriphos and cypermethrin) while resistance against deltamethrin was confirmed in LARA. HARA farms had a significantly higher prevalence of antibiotic residues (21.25%) in raw milk than in LARA (4%) farms (*p* <  0.05). Acaricide resistance and practice of reading drug use instructions were significantly associated with antibiotic residues in milk at farm level. Overall, the study provides vital information linking acaricide resistance to antibiotic use practices, consequently leading to antibiotic residues in milk.

## 1. Introduction

In recent times, tick acaricide failure or resistance has become one of the most important constraints to cattle production in Uganda. In southwestern Uganda, cases of *Rhipicephalus appendiculatus* and *Rhipicephalus* (*Boophilus*) *decoloratus* ticks that resist at least 2 classes of acaricides have been reported in over five districts [1]. Our previous study reported that farmers from southwestern Uganda rely exclusively on acaricides for tick control and did not practice integrated tick control approaches [2]. In addition, farms keeping crossbreeds of cattle were reported to be extensively using acaricides, shortening the application interval to twice a week and increasing concentration of acaricides by atleast two folds over manufacturer's recommended concentration as a strategy of addressing tick acaricide resistance [2, 3]. On the contrary, farmers from northwestern Uganda were found to be lacking knowledge on appropriate tick control and ocassionally used acaricides to control ticks, but their indigenous cattle were able to survive high tick burdens and associated tick-borne diseases (TBDs) [2]. Although farmers routinely apply acaricides on animals, the resistant ticks do not respond and continue to vector tick-borne pathogens causing an upsurge of TBDs [3]. As a result, farmers try to put in place measures to mitigate the associated TBDs of which use of antibiotics for both curative and prophylactic purposes remain very key [4].

Although the use of antibiotic drugs has signiﬁcantly improved the health and production efﬁciency of food-producing animals, antimicrobial residues in food animal products are increasingly raising public health concerns [5]. In circumstances, where drug withdrawal period is not observed or not precisely known by cattle keepers [6], drug residues are likely to contaminate milk. Some of the effects of antibiotic residues include selection of antibiotic resistant bacteria, which could later be transferred from animals to humans, through milk and its products. Moreover, the World Organization for Animal Health (OIE) reportedthat irrational use of antibiotics in food animal production is contributing to emergence of superbugs (strains of bacteria that are resistant to multiple antibiotics previously used to treat them), hence the need for more vigilance [7].

The increase in antibiotic therapy in cattle is presumed to lead to not only occurrence of antimicrobial residues in cattle products such as milk but also can cause emergence of antimicrobial resistance. Considering that veterinary drugs are mainly handled by unqualified farm personnel (farmers and herdsmen), especially in developing countries such as Uganda, this further facilitates misuse of antibiotics and mayaccelerate the development of antimicrobial resistance [8].

The purpose of this study was to assess and compare the farmers' practices regarding tick control and antibiotic usage on farms in areas of low and high tick acaricide resistance challenge in Uganda. Also, determine and compare the prevalence of antibiotic residues in milk from cows reared from the two different regions. Furthermore, the study established the relationship between acaricide failure and occurrence of antibiotic residues in milk and identified the potential risk factors associated with occurrence of antimicrobial residues in milk at farm level.

## 2. Materials and Methods

### 2.1. Study Area

**Figure 1:**
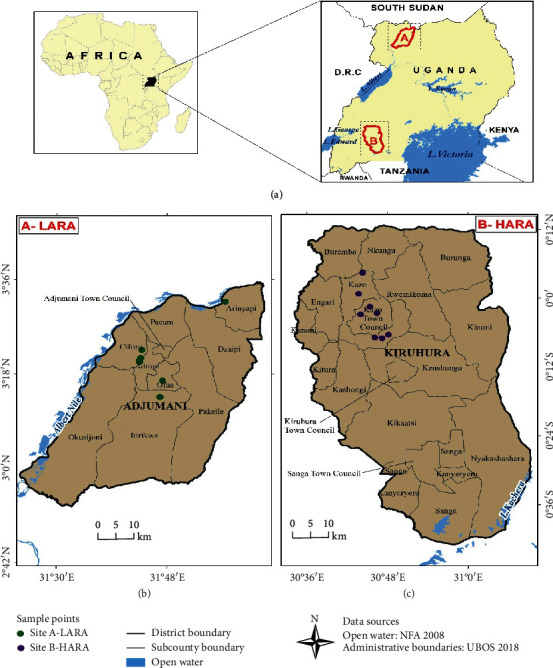
Map showing the study sites (LARA and HARA). The sampling points indicated on maps of both regions are less than ten (the actual sampling points) due to some sampling points being close to each other and appearing as one point on the map as a result of overlapping.

The study was conducted in two districts of Kiruhura and Adjumani representing the high acaricide resistance area (HARA) and the low acaricide resistance area (LARA) in southwestern and northern Uganda (Figure 1), respectively. Kiruhura district was selected because it is documented to be one of the hard-hit districts by acaricide resistance where multiacaricide resistance to all the 3 classes of acaricides, namely, organophosphates, amidines, and synthetic pyrethroids were detected. On the other hand, Adjumani district was reported to be free from acaricide resistance [1]. The cattle population in Adjumani district is estimated to be 220,000 while Kiruhura has approximately 340,000 cattle [9]. Majority of the cattle kept in Kiruhura district are crossbreeds of Ankole cattle and other exotic breeds, the common one being the Holstein Friesian breed [3]. On the other hand, small East African Zebu, Ankole cattle, Boran, and their crosses are the dominant breeds of cattle kept by farmers in Adjumani district. The crossbreeds kept in HARA are more susceptible to TBDs compared to thenative breeds kept in LARA, which are relatively more resistant to TBDs [3]. The communal grazing system is mainly practiced in LARA, whereas farmers from HARA practice the paddock system. The herd sizes vary in both regions depending on many factors such as land availability, breeds, and purpose for keeping cattle. However, farms located in LARA are organized into communal kraals with larger herd size (more number of cattle) compared to farms in HARA that are individually owned.

### 2.2. Study Design and Sample Size

This was a cross-sectional study, which involved collection of questionnaire data on tick control and antibiotic use at farm level, assessment of tick infestation on cattle, collection of individual cow composite milk samples, and collection of tick samples from randomly selected milking cows in a herd. A standard questionnaire was developed, pretested, and administered to farm owners in the local languages spoken in the two regions with the help of local extension officers. The questions captured the demographic characteristics of the respondents, practices of acaricide and antibiotic use, and ticks and TBD control in dairy cattle. A total of 10 farms were selected in each region (HARA and LARA) making the overall total of 20 farms in the two regions. From each farm, 10 milking cows were randomly selected to collect composite milk samples. A total of 200 composite milk samples were collected from the two regions with 100 samples being collected from each of the regions. Subsequently, half-body tick counts on each of the animals selected were performed. Tick samples were collected from the milking herd for culture and acaricide efficacy tests. Tick counts were performed on all the 200 milking cattle selected in the current study.

### 2.3. Sample Collection Procedure

#### 2.3.1. Tick Sample Collection and Half-Body Tick Counts

On each farm, 10 lactating cows were randomly selected and driven into an appropriate cattle crush to allow proper and humane handling of cattle. All visible ticks on one side of the body were counted using a tally counter and recorded. After tick counts, engorged ticks were collected from the same lactating animals for acaricide efficacy tests to establish the sensitivity of the ticks to common classes of acaricides namely organophosphates (chlorfenvinphos), amidines (amitraz), synthetic pyrethroid (deltamethrin), and coformulations (chlorpyriphos and cypermethrin). The ticks were placed in labeled perforated sample bottles to which a moist ball of cotton wool was added to regulate humidity for survival of ticks during transportation. The perforations were minute not to allow tick escape. The sample bottles were well secured with lids to prevent any escape of ticks. The sample bottles containing the ticks were placed in perforated baskets and transported to the Research Center for Tropical Diseases and Vector Control (RTC) laboratory at College of Veterinary Medicine, Animal Resources and Biosecurity (COVAB), Makerere University.

#### 2.3.2. Milk Sample Collection

The composite milk samples from each cow was collected from 10 cows per farm. Before taking the milk sample, the teats were cleaned with cotton wool soaked in 70% ethanol and wiped dry with a sterile paper towel. Hand milking was then performed to release the milk into a sterile 15 ml falcon tube labeled with the sample code, which included the cow identification, sample number, farm code, and date of collection. The sample tubes were transferred to a holding wrack inside a cool box containing ice packs for storage and transportation.

### 2.4. Tick Taxonomy and Culture

Ticks were microscopically identified to species level based on morphological features described by Walker and Bouattour [10]. Ticks were sorted to remove dead ones and skin debris. In addition, the different tick species from each farm were identified and sorted. For each tick population from a particular farm, three to four fully engorged ticks of the same species were placed in an incubation tube and labeled according to the species of the ticks, date of incubation, farm and district codes. The tube was then placed in an incubator at 27 ± 1°C and 80% relative humidity. The ticks were monitored daily to check for egg laying. After hatching, the larvae were kept in the incubator until they were 14 days old and used for acaricide efficacy tests as described by FAO [11].

### 2.5. Tick Acaricide Assay

A total of 7 tick populations from 7 farms were tested for acaricide susceptibility. Five tick populations (5 farms) were from the HARA while the rest were from the LARA. The manufacturers recommended concentration was considered as the diagnostic/discriminating dose for all the chemicals tested. However, one additional concentration level, which was twice the above dose, was also applied. The diluent used for all the acaricides was trichloroethylene and olive oil mixed in a ratio of 2 : 1 [11].The tests were conducted following the methods documented by Vudriko et al. [1]. The recommended (manufacturer) concentrations for deltamethrin, chlorfenvinphos, amitraz, and coformulated acaricides used in this study were 0.05 mg/ml, 0.5 mg/ml, 0.25 mg/ml, and 0.5 : 0.05 mg/ml, respectively. The corresponding double recommended concentrations were 0.1 mg/ml, 1 mg/ml, 0.5 mg/ml, and 0.1:1 mg/ml, respectively. Each tick population was subjected to both recommended and double recommended concentrations of all the four categories of acaricides.

Filter papers (Whatman No.1, Whatman, Madstone, United Kingdom) were used as a substrate for deltamethrin, chlorfenvinphos, and combination of chlorpyriphos and cypermethrin while Nylon fabric was used for amitraz. The filter papers were labeled, and 700 *µ*l of mixture (acaricide and diluent) was used to impregnate each filter paper or nylon fabric. Both filter papers and nylon fabric were left to stand in a fume hood for at least 2 hours to allow the evaporation of trichloroethylene. Each impregnated filter paper or nylon fabric was loaded with tick larvae (approximately 80 to 120 larvae) from the same farm and same species. The control groups were exposed to only the diluent. The packets were then secured with alligator clips and incubated at 29 ± 1°C and 80% relative humidity for 24 hours. Each experiment was carried out in duplicate. After 24 hours, the larval packets were removed in the order in which they were loaded in the incubator. The larvae were examined to identify and count the number of the dead and alive ticks in each packet. Mortalities were expressed as percentage of the total number of larvae exposed to the acaricide.

### 2.6. Antibiotic Residue Assay

Composite milk samples were used for screening of antibiotic residues using a standard broad-spectrum diffusion test called Delvotest® SP-NT test kit (DSM Food specialties B.V., The Netherlands). The test kit is made up of solid agar medium containing *Bacillus stearothermophilus varcalidolactis*, nutrients for growth, and bromocresol purple as an indicator. The tests were conducted following the instructions of the manufacturer as outlined in the insert protocol. Briefly, the water bath was preheated to 64 ± 2.0°C. Ampoules were removed individually from their wrack, and the aluminum foil covering the ampoule was removed carefully, 100 ul of homogenized milk sample was added, and the ampoule was labeled. Tests were performed in duplicates for comparison purposes and quality control. All test ampoules were covered with aluminum foil before being incubated for 3 hours at 64 ± 2.0°C in a preheated water bath. The result of this test was determined qualitatively using standard color changes in the 2/3 of the agar medium as follows: partially yellow was negative (the sample does not contain antibiotics or the antibiotics are below the detection sensitivity of the test), and completely purple was positive (the sample contains antibiotics at or above the detection sensitivity of the test).

### 2.7. Data Analysis

Data were entered into Microsoft Excel, sorted, cleaned, and later transferred to SPSS software version 23.0 (IBM SPSS Statistics for Windows, Version 23.0. Armonk, NY : IBM Corp.). Descriptive statistics from the data generated from the questionnaires were presented in a tabular format. The chi square test was performed to determine the factors associated with prevalence of antibiotic residues in milk for the two study sites. All variables were considered significant at *p* ≤ 0.05 at 5% level of significance.

### 2.8. Ethical Considerations

The study was approved by the institutional review board (No. VAB/REC/15/104) of the College of Veterinary Medicine, Animal Resources and Biosecurity, Makerere University. Ticks were handled under strict biosecurity measures involving restriction of access to tick incubation room and autoclaving all materials used for the larval packet test (LPT). Questionnaires were administered to only those participants who consented to the study, and the personal data of the individual respondents were kept conﬁdential. Milk and tick samples were collected from the cows following strict guidelines on humane handling of animals and respect for animal welfare.

## 3. Results

### 3.1. Demographics

A total of 20 respondents were interviewed with majority being male and had attained either primary or secondary education in both study sites (Table 1). All the farms selected in HARA kept crossbreeds of Ankole and exotic cattle 10 (100%), whereas small East African Zebu 9 (90%) was the dominant cattle breed kept in LARA. Half of the herds selected in HARA were small holder farms (<50 cattle) while the rest were large herds (>50 cattle). Whereas large herds dominated 9 (90%) in LARA, majority of the herds had more than 20 lactating cows in both study sites (Table 1).

### 3.2. Tick and Tick-Borne Diseases Control Practices

The tick control practices identified in HARA and LARA are shown in Table 2. Our study found that 100% and 80% of the respondents from the HARA and LARA, respectively, had visible ticks on their animals. All the farmers in HARA and LARA reportedly used acaricides for tick control. Compared to farmers (100%) in HARA who reported to have ever experienced the challenge of acaricide failure, none of the farmers from LARA had experienced that challenge. In addition, 30% and 100% of the farmers (respondents) reported that ticks would die whenever they applied acaricides on the animals in the HARA and LARA, respectively. Similarly, farmers in HARA reported a corresponding increase in the incidence of TBDs with an average of 3.2 cases treated per month per farm compared to an average TBD incidence of 0.2 cases per month per farm in LARA. The perceived percentage response of ticks to acaricides by farmers on the various farms in the HARA was estimated to be less than 80%, whereas in LARA, it was reported to be 100%. East Coast fever (ECF) was identified as the most common TBD in both HARA and LARA, though cases of coinfection (a combination of ECF, anaplasmosis, and babesiosis) were more common in the HARA than LARA. Half of the respondents in HARA reported a corresponding increase in the use of antibiotics on their farms as a result of perceived TBD challenge due to tick acaricide failure compared to the respondents from the LARA (Table 2). As regards to management of TBDs on the farm, use of commercial drugs was a common practice in both regions and heavily depended on the use of antibiotics such as oxytetracycline and a combination of penicillin and streptomycin with few variations. For example, antibiotics and buparvaquone were commonly used in HARA, whereas antibiotics and diminazene aceturate was commonly used in LARA for control of the various TBDs (Table 2).

### 3.3. Tick Count Results

The mean tick counts at individual farm level ranged from 0 to 18.6 and 0 to 12.8 in LARA and HARA, respectively, whereas the mean tick counts across farms was 5.95 (SD = 7.45; SEM = 2.36) and 4.17 (SD = 4.80; SEM = 1.52) for farms in LARA and HARA, respectively. There were no significant differences in mean tick counts between farms from LARA and those from HARA (*p* > 0.05). The common tick species observed on farms include *R.* (*B.*) *decoloratus* majorly in HARA, and *Rhipicephalus appendiculatus* was identified as a major tick species in farms located in LARA. Both *R. (B.) decoloratus* and *Rhipicephalus appendiculatus* were present in both regions. Also, to note is the presence of *Amblyomma variegatum* ticks in majority farms located in the LARA.

### 3.4. Tick Acaricide Susceptibility

Larvae of *R. (B.) decoloratus* ticks from 50% (*n* = 5) of the farms from HARA were tested against both the recommended and double recommended concentration of four different commercial acaricide formulations using LPT. The tick acaricide efficacy tests revealed that the ticks from farms located in the HARA were resistant to 3/4 classes of acaricides available on the Ugandan market. Noteworthy, the ticks collected from farms in HARA were 100% resistant to both amitraz and deltamethrin at the recommended and double recommended concentrations. Furthermore, ticks collected from farms located in HARA exhibited significant levels of resistance against coformulations (chlorpyriphos and cypermethrin) with a highest mean percentage mortality of 31.8% and 42.2% at recommended and double recommended concentrations, respectively. Only organophosphates (chlorfenvinphos) were fairly efficacious against ticks collected from farms from HARA with mean percentage mortalities ranging from 85.17 ± 3.07 to 93.51 ± 1.44 and 90.48 ± 1.19 to 97.06 ± 0.79 at recommended and double recommended concentrations, respectively (Table 3).

In the LARA, *Rhipicephalus appendiculatus* was tested for acaricide efficacy. Only 20% (*n* = 2) of the farms had test results since they were the only farms where engorged ticks were found. The test results reveal that the *Rhipicephalus appendiculatus* ticks tested from the LARA were 100% susceptible to amitraz and chlorfenvinphos at the recommended concentrations. The mean percentage mortality of these tick populations from LARA against coformulations ranged from 88.25 ± 3.06 to 97.53 ± 0.51, which was significantly higher than that observed with tick populations from HARA. Unexpectedly, *R. appendiculatus* ticks from LARA showed resistance to deltamethrin, and the mean percentage mortality ranged from 18.26 ± 3.97 to 35.97 ± 4.46 at recommended concentration (Table 4).

### 3.5. Disease Occurrence and Antibiotic Use Practices on Farms

The mean number of lactating cows on the farms in HARA (mean = 19.8; SD = 7.21; SEM = 2.28) was lower than that in LARA (mean = 24.7; SD = 17.8; SEM = 5.60). Disease incidence among milking cows was common in HARA with all farmers reporting an average of 5.9 cows (SD = 8.67; SEM = 2.74) falling sick per farm in a period of three months compared to an average of 0.7 (SD = 0.82; SEM = 0.26) animals falling sick per farm in the same period in LARA. The mean number of cases treated per month following acaricide failure was 2.9 in HARA (SD = 1.52; SEM = 0.48) compared to none in LARA.

ECF, coinfections of TBD, and mastitis were among the diseases that the farmers reported to be commonly affecting the milking herd in majority of the farms in HARA. In addition to the above diseases, black quarter and trypanosomiasis were among the common diseases reported to be affecting lactating cows in LARA (Table 5). The study reveals that 90% of the diseases were self-diagnosed by farmers and herdsmen in both HARA and LARA. Generally, antibiotics and buparvaquone were the commonly used veterinary drugs on the farms visited in both regions. Every farm visited in the HARA routinely used antibiotics for control of diseases especially TBDs and mastitis. Fifty percent of the farms in HARA also used a combination of antibiotics and buparvaquone to control diseases among the milking cattle. The frequency of antibiotic use on the farm was reported to be either weekly (40% and 40%) or monthly (50% and 40%) in the HARA and LARA, respectively. Oxytetracycline (100%) was the most commonly used antibiotic on farms visited. Antibiotics and antiparasitic drugs (imidocarb dipropionate, anthelmintic, and acaricides) were heavily stocked at the farm for emergencies in both regions. None of the farms that participated in this study kept records of treatment of animals in both regions. On assessing whether farmers followed and respected the withdrawal period for the antibiotics they routinely used to treat the milking herd, we found that a majority (8/10 and 7/10 for HARA and LARA, respectively) of the farmers did not follow the withdrawal period and continued to milk their cows normally after treatment. Farmers (100%) in HARA reported to have always read the withdrawal period on the drug label compared to the majority (80%) of farmers in the LARA who reported to have never read drug labels to understand the withdrawal period of the drugs they used. Further probing as to why some farmers could not continue to milk the treated cows revealed that only one farmer from HARA was following the withdrawal period as indicated on the drug label. The other farmers gave other reasons such as the udder being inflamed and could not be milked, milk being unsafe, and fear of milk containing antibiotic residues. A majority of the milk from treated cows in HARA and LARA regions ended up on the market (40% and 50%, respectively). The rest of the milk from treated cows was used for home consumption and making other dairy products, while some farmers reported that such milk could be given to dogs. The majority of the farmers from HARA (90%) and LARA (70%) accessed their drugs including antibiotics from the local drug shops. Correspondingly, majority of the farmers from both regions obtained advice on animal health from drug shop attendants regardless of their qualifications. Furthermore, some farmers reported that they obtain advice on animal health from fellow farmers. Estimation of amount of the antibiotic to be administered to a sick cow was the most common method used by farmers to determine the dose for a given animal (Table 6). In the process of estimation of the dose to administer to a sick cow, majority of the farmers in both regions considered either the severity of the disease or the estimated body weight of the animal. On further investigation, it was found that, out of those farmers who took time to read drug labels in both HARA and LARA, much emphasis was put on understanding the dosage rate of the drug with farmers in the HARA additionally looking at the expiry date of the drug. Some farmers reported to be using antibiotics for prophylactic purposes in both regions. Particularly, oxytetracycline was identified as the most commonly used antibiotic on farms for prophylactic purposes (Table 6).

### 3.6. Antibiotic Residues in Milk

**Figure 2:**
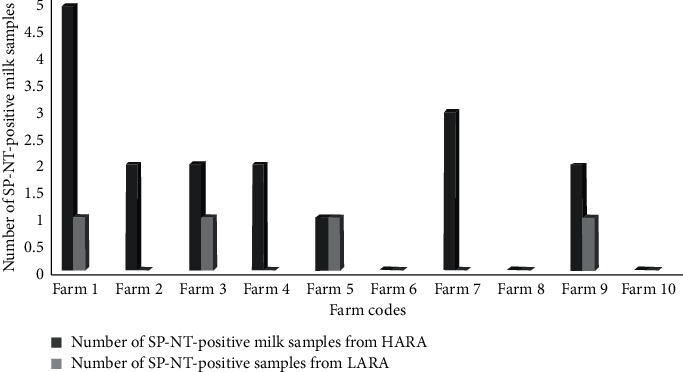
Comparative presentation of the level of antibiotic residues in milk on farms in the two study regions (HARA and LARA). The paired farms are independent of each other.

At farm level, 7/10 and 4/10 of the farms in HARA and LARA, respectively, had at least one cow positive for antibiotic residues in milk. The overall prevalence of antibiotic residues in milk at farm level was significantly different (*p* < 0.05) in the two regions. Farms from HARA had higher overall prevalence (21.25%) compared to 4% obtained from farms located in LARA. Comparing the individual farm prevalence between the two regions, milk from farms located in HARA continued to consistently have higher prevalence of antibiotic residues compared to their counterparts in the LARA as shown in Figure 2. The highest prevalence of antibiotic residues in milk at individual farm level was 5/10 (mean prevalence = 2.13; SD = 1.57) in one of the farms located in HARA, whereas the highest was 1/10 (mean prevalence = 0.40; SD = 0.512) in LARA.

### 3.7. Risk Factors Associated with Occurrence of Antibiotic Residues in Milk (SP-NT Positive Result)

The practice of reading instructions for use (drug insert or drug label) of the various antibiotic drugs on the farm was significantly associated with antibiotic residues in milk (*p*=0.007) at 5% level of significance. Furthermore, milk from farms where respondents reported to be facing the challenge of acaricide resistance was more likely to contain antibiotic residues (85.71%) compared to the milk obtained from farms where respondents reported that ticks would die after acaricide application where only 38.46% had antibiotic residues in milk (*p*=0.043) at 5% level of significance. On the other hand, the chance of milk from crossbreed of exotic cattle having antibiotic residues was higher (72.7%) compared to milk obtained from small East African Zebu cattle where only 33.3% of the milk had detectable levels of antibiotic residues, though it was not statistically significant at 5% level of significance (*p*=0.078). Prophylactic use of antibiotics in dairy cattle was marginally associated (*p*=0.064) with antibiotic residues in milk at farm level (Table 7).

## 4. Discussion

Acaricide resistance and irrational use of antibiotics in food animals are a global concern because they threaten the fundamentals of animal and human health. This phenomenon is evident in Uganda where reports have shown that acaricide resistance leads to more cases of TBDs including East Coast fever, babesiosis, anaplasmosis, and ehrlichiosis [3]. As a result, the farmers use more antibiotics and antiprotozoal drugs, which may leave residues in milk. Therefore, this study was conducted to compare on-farm practices regarding tick control and antibiotic use and further assess the level of antibiotic residues in milk in areas of low and high acaricide resistance challenge in Uganda.

As regards demographics, majority of the respondents in both regions were male and were fairly literate with most having capacity to read and write. Farms located in HARA kept mainly crosess of exotic dairy cattle [2, 12]. Indigenous dual-purpose cattle were the most dominant breed kept in the LARA. HARA was characterized with fairly small- to medium-sized dairy cattle herds kept under the paddock system, whereas large herds kept on communal grazing lands were common in LARA. The large herds are supported by the practice of clan ownership of cattle and communal grazing practice in the area [2]. Also, since the dual-purpose indigenous cattle are less productive in terms of milk production, farmers may be forced to keep large numbers of animals in order to compensate for their inherent low milk production and consequently increase the overall farm productivity. On the other hand, farmers in HARA kept small herds of dairy breeds due to individual ownership of cattle and limited land resulting from land fragmentation coupled with mixed farming where the available land is shared between livestock and crop farming. Since the productivity of the breeds kept in HARA is fairly high, the farmers in HARA may have the freedom to keep a few high producing cows on the farm. Other factors such as limited cattle feeds and water may also affect the herd size in HARA.

Acaricide application was the main method used to control ticks in both regions, though there were varying reports of tick response to acaricides. Use of acaricides to control ticks by majority farmers has also been reported in other countries within the East African region especially Tanzania [13]. The reported reduction in response of ticks to acaricides in HARA region is consistent with the previous reports, which confirmed presence of super resistant ticks in the region [1, 14]. The report further indicates that ticks from the districts located in HARA had developed multiple resistance and could resist at least two classes of acaricide molecules. The same report highlighted that ticks collected from districts located in LARA were susceptible to all acaricides available on the market. High TBD incidences of up to an average of 3.2 cases per month per farm were reported in farms located in HARA compared to very low TBD cases reported in farms located in LARA. The increase in TBD cases is attributed to reduced response of ticks to acaricides in HARA region. This is further explained by the hypothesis that whenever there is an increase in the population of resistant ticks, there may be a corresponding increase in the incidence of diseases vectored by these particular ticks. It was not surprising to find that ECF was the most common TBD in both regions. This is in agreement with previous reports, which found a prevalence of over 90% of *Theileria parva* in bovine samples submitted for clinical examination at Veterinary Central Diagnostic Laboratory at Makerere University [15]. Another study by Muhanguzi et al. [16] also emphasized the veterinary importance of ECF in cattle. Similarly, ECF was ranked as the most common TBD by farmers in a study conducted in Tanzania [13]. Furthermore, farms located in HARA had higher cases of other TBDs such as anaplasmosis and babesiosis compared to farms from LARA. This is explained by the vector tick presence in the region. *R. (B.) decoloratus* was the dominant tick species in HARA while *Rhipicephalus appendiculatus* dominated the farms in LARA. This is in agreement with a study which highlighted the same tick species as the major ones in the same regions [2]. Previous studies have found a positive relationship between the presence of a given tick species and the prevalence of associated TBD [17]. On the other hand, farmers from LARA reported cases of heartwater, which is associated with the presence of *Amblyomma* ticks, which vector the causative agent (*Ehrlichia ruminantium*) of heartwater. The absence of *Amblyomma* ticks on farms in HARA could be attributed to heavy use of acaricides over a long period of time. This further explains why farmers in HARA did not report any cases of heartwater. It was noted that management of TBD cases in both regions relied on use of antibiotics. On top of using antibiotics, farms in HARA also used drugs such as buparvaquone and parvaquone routinely in the control of TBDs. Antitrypanosome drugs such as diminazene aceturate was reported to being used by farmers to control TBDs in LARA. LARA is located in one of the tsetse fly-infested regions of Uganda [18], and the cattle have high chances of being exposed to African Animal Trypanosomiasis (AAT). This explains the rampant use of diminazene aceturate, which is normally a curative drug for AAT.

Tick infestation on cattle was not significantly different in the two areas. The wide spread of tick resistance in HARA explains the reported level of tick infestation on cattle, whereas the moderately high tick infestation in LARA was due to haphazard and inconsistent application of acaricides as noted by Vudriko et al. [1]. Absence of ticks on some cattle in the HARA may not necessarily reﬂect acaricide eﬀectiveness but may also be due to excessive use of acaricides coupled with use of agrochemicals to eliminate resistant ticks. Majority of the farmers in LARA are subsistence farmers and sometimes may not afford regular purchase of acaricides for regular tick control [1].

The tick susceptibility to acaricides was determined against the 4 acaricide molecules namely chlorfenvinphos, deltamethrin, amitraz, and a combination of chlorpyriphos and cypermethrin. Super resistant *R. (B.) decoloratus* ticks were detected and confirmed in HARA, which is consistent with previous studies conducted by researchers from Makerere University [14] where multiacaricide resistant *R. (B.) decoloratus* ticks were reported from the same region. However, the acaricide susceptibility results for ticks collected from LARA were different from that published in a study by Vudriko et al. [19], which reported that farms from LARA were free of tick resistance to all classes of acaricides. However, the current study has revealed that ticks from this region were slowly developing resistance to deltamethrin though still susceptible to the rest of the classes of acaricides. The observed resistance against deltamethrin in LARA was likely due to promotion and prolonged use of synthetic pyrethroids against ticks, nuisance flies, and tsetse flies by the district veterinary department. In addition, pyrethroids are packaged in small quantities that are affordable to most farmers in LARA who dominantly practice subsistence farming.

Disease incidence among milking cows was higher in farms located in HARA with an average of six cases per farm in a period of just three months compared to LARA where farmers hardly reported an average of one case per farm. Disease incidence influences the use of antibiotics on the farm [20]. In both regions, ECF and coinfections of TBDs and mastitis were reported as the most common diseases affecting lactating cattle. This is further supported by previous published reports, which have consistently identified ECF as the most common disease affecting cattle in Uganda and neighboring countries [8, 15, 21]. Farms located in HARA were found to be practicing self-diagnosis and self-treatment of animal diseases without consulting the qualified veterinarian, and this was not any different in farms located in LARA. This corroborates well with the findings of a study conducted in Ngoma, Nakaseke district (Central Uganda), which reported that 97.4% of the farmers treated the sick animals themselves while only 2.6% endeavored to find a veterinarian to handle the treatment of the sick animals on the farm [8]. This may also explain the rampant misuse and irrational use of antibiotics for the prevention and control of unconfirmed diseases on farms located in HARA. The frequent use of other drugs other than antibiotics such as buparvaquone for the control of TBDs was observed in the HARA region and very few farms in the LARA region. This was attributed to an increased incidence of TBDs as a result of acaricide failure due to tick resistance in the region. Since majority of the farmers in HARA keep exotic cattle and their crosses, which are more susceptible to TBDs [3], that creates more reasons for farmers to use acaricides and antibiotics more often compared to farmers in the LARA who keep indigenous cattle, which are quite resistant and tolerant to TBDs. Due to high TBD incidence reported by farmers in the HARA, farmers may be forced to stock and create mini veterinary drug stores at the farm to facilitate early intervention and for prophylactic purposes. This practice also promotes imprudent behaviors especially self-diagnosis and self-medication by farmers. The veterinary drugs stocked by most farmers in the HARA include antibiotics, anthelmintic, acaricides, and buparvaquone, whereas the farms in LARA stock antibiotics and acaricides. Among the antibiotics, oxytetracycline was the most commonly used antibiotic in both regions. This is likely to be associated with its recommendation in the treatment of TBDs such as ECF and anaplasmosis. Previous studies have reported about oxytetracycline being the most commonly used antibiotic on farms both in Uganda and Tanzania [8, 21].

It is surprising that though all the respondents from the HARA reported to read drug labels and were aware of the withdrawal period, majority (80%) did not observe the withdrawal period for the antibiotics they used routinely on the farm. A similar finding was reported by a study conducted in Central Uganda [8]. As a result, there are high chances of products such as milk produced by these farms to contain drug residues especially antibiotic residues. This may act as a precursor for development of antibiotic resistance in animal and human populations. Furthermore, failure to respect the withdrawal period may lead to production losses since antibiotic residues interfere with starter cultures leading to production of poor-quality yoghurt. This practice was further complicated by the absence of treatment records on all farms in both regions. This made it difficult to trace and identify the treated cows for purposes of making sure that only safe milk was delivered to the market for human consumption.

The study reports that much of the milk from treated cows where the withdrawal period had not been observed ended up on market in both regions. This suggests that the consumers could be at risk of continuous exposure to subtherapeutic levels of antibiotics through routine consumption of contaminated milk and associated products. The risk could be much higher in HARA since majority of the farms in that region were heavy users of antibiotics in dairy animals. Farmer ignorance about the importance of strictly observing the withdrawal period, the practice of seeking advice from unqualified personnel, uncontrolled access to medicines without prescriptions, stocking of veterinary drugs at farms, and the practice of self-diagnosis and self-medication could be some of the factors promoting noncompliance to withdrawal periods by farmers. In addition, weak food safety regulatory frameworks coupled with a weak veterinary drug and veterinary practice regulation in the country may be contributing to the problem. The national action plan on antimicrobial resistance for Tanzania also identified noncompliance to withdrawal periods in animals as one of the drivers of AMR [22]. Mere estimation of dose and dosage of antibiotics to be administered to sick lactating cows in both regions is suggestive of rampant misuse of antibiotics through either over dosing or under dosing [23], which can be a precursor of antibiotic resistance development. In another study, 100% of the farmers who practiced self-medication of their animals were found to only estimate the weight of animals prior to drug administration [8].

The prevalence of antibiotic residues was significantly high in milk collected from cows in HARA compared to that collected from LARA. The prevalence of antibiotic residues in individual cow milk in HARA farms was high at 21.25%. This finding is comparable with other studies conducted in Kenya, which revealed a slightly higher prevalence of 24% from milk vending machines samples, and 24% of street vendor samples were presumably positive for at least one antibiotic [24]. The slight difference in prevalence may be attributed to differences in samples collected. The study conducted in Kenya considered bulk milk samples where the chances of a few individual cow milk containing antibiotic residues contaminating the whole pool of milk was high and hence increasing chances of positive results. On the other hand, our study considered individual cow milk as the smallest unit of sampling, and therefore, there were no chances of cross contamination. Studies conducted in Tanzania reported that 36% of marketed milk samples were found to contain antibiotic residues [6]. The high prevalence of samples containing antibiotic residues in the study conducted in Tanzania was attributed to three factors: mastitis therapy, treatment of vector borne diseases, and direct addition of antibiotics for preservation of milk in the absence of refrigeration. In another related study conducted in Kuwait, it was found out that 29.1% of the local fresh milk samples contained antibiotic residues far above the maximum residue level (MRL) for tested residues with tetracycline as predominant residue [5]. The observed prevalence of antibiotic residues in milk from HARA compared to LARA may be associated with heavy and nonselective use of antibiotics for both curative and prophylactic purposes due to high TBD incidence linked to high acaricide resistance challenge in the region and noncompliance to withdrawal periods. The breed-related susceptibility of cattle to tropical diseases [25] might also account for the observed difference in antibiotic use patterns and consequently antibiotic residues prevalence in milk in the two contrasting regions. This study, therefore, is reporting for the first time a positive relationship between tick acaricide resistance and antibiotic residue prevalence in milk. Areas that are hard-hit by acaricide resistance could be at risk of producing milk contaminated with antibiotic residues, and the public could be at risk of consuming contaminated milk and milk products.

Regarding the risk factors associated with presence of antibiotic residues in milk at farm level, the study revealed that tick resistance to acaricides and the practice of reading instructions for use on the drug labels were significantly associated with presence of antibiotic residues in milk. Acaricide resistance may lead to an upsurge of TBD cases on dairy farms especially those keeping exotic crosses of cattle, which are more susceptible to TBDs [3]. Consequently, it leads to heavy use of antibiotics for both curative and prophylactic purposes against TBDs and in the process promoting irrational use of antibiotics. This leads to milk contamination with antibiotic residues. Continuous exposure of bacterial pathogens especially mastitis-causing bacteria to sublethal doses of antibiotics in milk could accelerate the development of antibiotic resistance. The finding that milk from farms where respondents reported to have always been reading the instructions for use and withdrawal periods being more likely to contain antibiotic residues is quite puzzling. However, this may be attributed to pseudoconfidence developed by farmers over time as they have always been easily accessing and using antibiotics on the farm. In addition, there is a likelihood that though they claim to read the information on the drug labels, there are high chances that they do not understand the information. This may be due to either because of the technical language used or they do not read all the information written on the drug labels. The above finding may be further explained by the fact that literate farmers are likely to have formal employment plus more than one source of income, and hence able to buy much more antibiotics for use at farm level compared to their illiterate counterparts whose purchasing power is likely to be low due to limited incomes. Other studies conducted in the region reported the following risk factors: lack of understanding of risks related to antibiotic contamination of food, poor or no treatment records, and lack of a monitoring system are major risks for contamination of milk on small-scale farms in Kenya [26]. It is important to note that this is the first study to report acaricide resistance as a risk factor associated with presence of antibiotic residues in milk at farm level in Uganda. Due to budgetary limitations, the sample size was not adequate to statistically identify and document all the on-farm risk factors associated with occurrence of antibiotic residues in milk.

## 5. Conclusions

Overall, this study has provided an insight into the link between tick control failure/acaricide resistance and antibiotic use practices contributing to the occurrence of antibiotic residues in milk. Acaricide resistance leads to heavy use of antibiotics to control the associated TBDs, thereby, increasing the chances of milk being contaminated with antibiotic residues. The findings of this study should stimulate stakeholder dialogue on promoting rational use of veterinary antibiotics and the need for promoting effective tick control strategies to prevent the possible ramification of negative effects of tick control failure on public health. There is a need to include antibiotic residue testing among the commonly performed tests for milk quality assessment in Uganda to protect the public from chronic exposure to subtherapeutic levels of antibiotics through consumption of milk and related products.

## Figures and Tables

**Table 1:** Demographics in the two study sites of HARA and LARA.

Variable	Category	Frequency (%)	
		HARA	LARA
Sex of respondents	Male	7 (70)	8 (80)
	Female	3 (30)	2 (20)
Level of education	Illiterate	1 (10)	3 (30)
	Primary	3 (30)	3 (30)
	Secondary	4 (40)	4 (40)
	Diploma	1 (10)	0
	University	1 (10)	0
Breed of cattle	Crossbreeds of Ankole and exotic breeds	10 (100)	1 (10)
	Small East African Zebu	0	9 (90)
Herd size	0–20	0	0
	21–50	5 (50)	1 (10)
	>50	5 (50)	9 (90)
Number of lactating cows	1–10	1 (10)	3 (30)
	11–20	5 (50)	1 (10)
	21–30	4 (40)	5 (50)
	Above 30	0	1 (10)

*N* = 10 for both HARA and LARA.

**Table 2:** Tick control practices in the two study sites.

Variable	Category	Frequency (%)	
		HARA	LARA
Presence of ticks on the cattle	Yes	10 (100)	8 (80)
	No	0	2 (20)
Presence of acaricide failure challenge on the farm	Yes	10 (100)	0
	No	0	10 (100)
Methods used to control ticks	Use of acaricides	10 (100)	10 (100)
	Herbs	0	0
Whether ticks die after spraying/dipping or not.	Yes	3 (30)	10 (100)
	No	7 (70)	0
Estimate of response rate of ticks to acaricides by farmers	25%	1 (10)	0
	50%	5 (50)	0
	75%	4 (40)	0
	100%	0	10 (100)
Increase in incidence of TBD following acaricide failure	Yes	10 (100)	1 (10)
	No	0	9 (90)
Tick-borne diseases commonly treated on the farm	ECF	4 (40)	10 (100)
	Coinfections of TBDs	6 (60)	0
Estimated number of cases treated per month following acaricide failure	1–2	3 (30)	1 (10)
	3–5	6 (60)	0
	Above 5	1 (10)	0
	Not applicable	0	9 (90)
Common drugs used to treat the tick-borne diseases	Antibiotics	0	7 (70)
	Antibiotics and diminazene aceturate	0	3 (30)
	Antibiotics and buparvaquones	10 (100)	0
Any increase in frequency of cattle treatment with antibiotics following acaricide failure	Yes	5 (50)	0
	No	5 (50)	0
	Not applicable	0	10 (100)
Prophylactic use of antibiotics on farms	Yes	3 (30)	8 (80)
	No	7 (70)	2 (20)
Commonly used antibiotic for prophylactic purposes in cattle	Oxytetracycline	3 (30)	10 (100)
	Not applicable	7 (70)	0

*N* = 10 for both HARA and LARA.

**Table 3:** Acaricide efficacy results for *R. (B.). decoloratus* ticks collected from HARA.

Farms	Percentage mortality per the different acaricides tested (mean ± SEM)							
	Deltamethrin		Coformulation		Chlorfenvinphos		Amitraz	
	0.05 mg/ml	0.1 mg/ml	0.55 mg/ml	1.1 mg/ml	0.5 mg/ml	1 mg/ml	0.25 mg/ml	0.5 mg/ml
KK-08	0	0	3.09 ± 0.06	3.85 ± 0.18	86.08 ± 2.4	93.67 ± 1.1	0	0
KK09	0	0	5.32 ± 0.32	7.99 ± 1.03	85.17 ± 3.0	90.77 ± 0.2	0	0
K06	0	0	13.78 ± 1.9	36.70 ± 7.8	93.51 ± 1.4	96.18 ± 1.1	0	0
K05	0	0	7.10 ± 1.17	42.2 ± 3.67	90.63 ± 1.2	97.06 ± 0.7	0	0
K03	0	0	2.26 ± 0.63	8.84 ± 0.38	85.19 ± 2.2	90.48 ± 1.1	0	0

*N* = 5.

**Table 4:** Acaricide susceptibility results for *R. appendiculatus* collected from LARA.

Farm	Percentage mortality per the different acaricides tested (mean ± SEM)			
	Deltamethrin (0.05 mg/)	Chlorfenvinphos (0.5 mg/ml)	Coformulation (0.55 mg/ml)	Amitraz (0.25 mg/ml)
A002	18.26 ± 3.97	88.25 ± 3.06	100 ± 0.00	100 ± 0.00
A005	35.97 ± 4.46	97.53 ± 0.51	100 ± 0.00	100 ± 0.00

*N* = 2.

**Table 5:** Practices, attitudes, and knowledge of farmers regarding antibiotic use in lactating cows.

Variable	Category	Frequency (%)	
		HARA	LARA
Whether sick cows were observed among the milking herd in the last three months	Yes	10 (100)	5 (50)
	No	0	5 (50)
Name of disease treated in the last three months	Black quarter	0	1 (10)
	Mastitis	1 (10)	0
	Coinfections of TBDs	6 (60)	1 (10)
	ECF	3 (30)	2 (20)
	Trypanosomiasis	0	1 (10)
	None	0	5 (50)
Person responsible for disease diagnosis	Farmer	8 (80)	5 (50)
	Fellow farmer	0	3 (30)
	Herdsman	1 (10)	1 (10)
	Veterinary doctor	1 (10)	1 (10)
Drugs used to treat sick cows	Oxytetracycline	2 (20)	7 (70)
	Penicillin and streptomycin	0	1 (20)
	Tylosin	0	1 (10)
	Gentamicin	1 (10)	0
	Oxytetracycline and buparvaquone	7 (70)	0
Person administering drugs	Farmer	6 (60)	9 (90)
	Paraveterinarians	2 (20)	1 (10)
	Veterinarian	2 (20)	0
Category of drugs commonly used to treat sick dairy cows on the farm	Antibiotics	5 (50)	10 (100)
	Antibiotics and buparvaquones	5 (50)	0
Frequency of antibiotic use	Daily	1 (10)	2 (20)
	Weekly	4 (40)	4 (40)
	Monthly	5 (50)	4 (40)
Concentration (%) of oxytetracycline normally used	10% oxytetracycline	10 (100)	3 (30)
	12.5% oxytetracycline	0	2 (20)
	20% oxytetracycline	0	5 (50)
Drugs usually kept at the farm for emergencies (multiple options available)	Antibiotics	10 (100)	10 (100)
	Acaricides	10 (100)	10 (100)
	Dewormers	10 (100)	7 (70)
	Imidocarb dipropionate	10 (100)	2 (20)
	Buparvaquones and parvaquones	10 (100)	0
Availability of treatment records	Yes	0	0
	No	10 (100)	10 (100)
Whether the farmer continued to milk the cows or not immediately after treatment with drugs.	Yes	8 (80)	7 (70)
	No	2 (20)	3 (30)

*N* = 10 for both HARA and LARA.

**Table 6:** Practices, attitudes, and knowledge of farmers regarding antibiotic use in lactating cows.

Variable	Category	Frequency (%)	
		HARA	LARA
Fate of milk obtained from treated cows	Home consumption	1 (10)	2 (20)
	Market	4 (40)	5 (50)
	Given to dogs	1 (10)	0
	Making other dairy products	1 (10)	0
	Not applicable	3 (30)	3 (30)
Number of withdrawal days observed	1 to 3 days	3 (30)	0
	Not applicable	7 (70)	10 (100)
Reason for stopping milking the animal after treatment	Milk has antibiotic residues	1 (10)	0
	Milk is not safe	1 (10)	0
	Udder was inflamed	1 (10)	0
	Not applicable	7 (70)	10 (100)
Whether or not they read the withdrawal period on the label	Yes	10 (100)	0
	No	0	8 (80)
	Not applicable	0	2 (20)
Source of drugs used on the farm	Local vet drug shop	9 (90)	7 (70)
	Vet pharmacy	1 (10)	3 (30)
Advice seeking	Yes	10 (100)	7 (70)
	No	0	3 (30)
Source of advice	Drug shop attendant	8 (80)	4 (40)
	Fellow farmer	1 (10)	2 (20)
	Vet doctor	1 (10)	1 (10)
	Not applicable	0	3 (30)
Method of determining the right dose to administer	Calculate the dose	0	1 (10)
	Consult a vet	2 (20)	1 (10)
	Estimation	5 (50)	7 (70)
	Reading drug labels	3 (30)	1 (10)
Factors considered in determining the dose of antibiotic to give to a sick cow	Dosage rate	1 (10)	1 (10)
	Severity of the disease	4 (40)	2 (20)
	Weight of the animal	4 (40)	6 (60)
	Age of animal	1 (10)	0
	Not applicable	0	1 (10)
Whether farmers read drug labels or not	Yes	10 (100)	3 (30)
	No	0	7 (70)
Emphasis while reading the instructions on the drug labels	Dosage rate	2 (20)	6 (60)
	Expiry date	0	3 (30)
	Withdrawal period	0	0
	Age of the animal	2 (20)	0
	Contraindications	0	0
	Ingredients	0	0
	Severity of the disease	4 (40)	0
	Body weight	4 (40)	0
	Not applicable	0	1 (10)

*N* = 10 for both HARA and LARA.

**Table 7:** Risk factors associated with antibiotic residues in milk from farms in HARA and LARA.

Variables	Category	SP-NT result		Pearson value (X^2^)	df	*p* value
		Negative	Positive			
	Female	0	5			
Breed of cattle	Crosses	3 (27.3%)	8 (72.7%)	3.1038	1	0.078^*∗*^
	Small East African Zebu	6 (66.7%)	3 (33.3%)			
Whether farmers read drug labels or not	Yes	3 (23.08%)	10 (76.92%)	1.6345	2	0.007^*∗∗∗*^
	No	6 (85.71%)	1 (14.29%)			
Whether ticks die after spraying/dipping or not	Yes	8 (61.54%)	5 (38.46%)	4.1048	1	0.043^*∗∗*^
	No	1 (14.29%)	6 (85.71%)			
Prophylactic use of antibiotics on farms	Yes	7 (63.64%)	4 (36.36%)	0.6061	2	0.064^*∗*^
	No	2 (22.22%)	7 (77.78%)			
	N/A	2 (28.57%)	5 (71.43%)			

^*∗∗∗*^, ^*∗∗*^, and ^*∗*^ means 1%, 5%, and 10% level of significance.

## Data Availability

The data used to support the findings of this study are included within the article.
